# Optimization of Extraction of Bioactive Peptides from Monkfish (*Lophius litulon*) and Characterization of Their Role in H_2_O_2_-Induced Lesion

**DOI:** 10.3390/md18090468

**Published:** 2020-09-17

**Authors:** Xiaoxiao Tian, Jiawen Zheng, Baogui Xu, Jiena Ye, Zuisu Yang, Falei Yuan

**Affiliations:** Zhejiang Provincial Engineering Technology Research Center of Marine Biomedical Products, School of Food and Pharmacy, Zhejiang Ocean University, Zhoushan 316022, China; TIANXIAOXIAO0208@163.com (X.T.); jwzheng1996@163.com (J.Z.); xubaogui96@163.com (B.X.); yjn4806@163.com (J.Y.)

**Keywords:** monkfish (*Lophius litulon*), <1 kDa peptides, response surface methodology, antioxidant activity

## Abstract

Background: Marine fish meat has been widely used for the extraction of bioactive peptides. This study was aimed to optimize the preparation of monkfish muscle peptides (LPs) using response surface methodology (RSM) and explore the antioxidant activities of <1 kDa LPs. Methods: Peptides were prepared from the muscles of monkfish (*Lophius litulon*), and five proteases were tested to hydrolyze muscle proteins. The hydrolysate that was treated using neutrase showed the highest degree of hydrolysis (DH) and 1,1-diphenyl-2-picrylhydrazyl (DPPH) scavenging activities. Results: The optimized conditions were as follows: water/material ratio of 5.4:1, a time span of 5 h, pH of 7.0, enzyme concentration of 2000 U/g, and temperature of 45 °C; the maximum DPPH scavenging activity and DH were 92.861% and 19.302%, respectively. LPs exhibited appreciable antioxidant activities, including DPPH radical, hydroxyl radical, 2,2′-azinobis-3-ethylbenzthiazoline-6-sulphonate (ABTS) radical, and superoxide anion scavenging activities. LPs attenuated H_2_O_2_-related oxidative injury in RAW264.7 cells, reduced the reactive oxygen species (ROS) and malondialdehyde (MDA) levels, and increased the superoxide dismutase (SOD), glutathione peroxidase (GSH-Px), and catalase (CAT) levels. Conclusion: We concluded that LPs could be an ideal source of bioactive peptides from monkfish and also have pharmaceutical potential.

## 1. Introduction

In recent years, natural antioxidants have received growing attention with regard to their free radical scavenging property and their ability to slow down disease progression [1]. Oxidation is a key process in all living animals despite the side effect of generating free radicals [2]. These radicals are so unstable that they give rise to cell lesions and various diseases [3]. Antioxidants that are synthesized by chemical methods may have adverse effects on humans; therefore, the development of safe and effective natural antioxidants is urgently needed [4,5]. Antioxidant peptides that are released by enzymatic hydrolysis are safe and have low molecular weight and strong antioxidant activities [6]. Marine bioactive peptides are a group of proteins that have critical functions in host defense systems of marine organisms. They are now being widely used in drug research and development as antibacterial, antiviral, and anticancer agents [7]. A number of studies have used the hydrolysates of fish meat as antioxidant peptides, such as peptides from eel, cod, tuna, salmon, and croaker [8,9,10,11,12]. Monkfish (*Lophius litulon*) is distributed in the western part of the North Pacific Ocean and the coastal waters of China. Generally, large quantities of monkfish are exported as aquatic products for human consumption; however, smaller monkfish is manufactured as fish meal.

Response surface methodology (RSM) is a useful tool for the evaluation of independent parameters to modify experimental variables [13]. In recent years, RSM has been applied to the extraction of fish protein hydrolysates and peptides, such as yellowfin tuna (*Thunnus albacares*) [14], stonefish [15], halibut [16], and red tilapia (*Oreochromis* spp.) [17], as well as monkfish [18,19]. The preparation process has yet to be optimized.

In this study, hydrolytic effects of five enzymes were studied, and neutral protease was identified as the optimal enzyme to hydrolyze monkfish muscle. RSM was used to modify the process of purification. The molecular weight (MW) distribution of monkfish muscle polypeptide was obtained using high-performance liquid chromatography (HPLC). Monkfish muscle peptides with MW <1 kDa were prepared using ultrafiltration and freeze-drying followed by the determination of its antioxidant capacity.

## 2. Results and Discussion

### 2.1. Selection of the Optimal Enzyme

**Figure 1:**
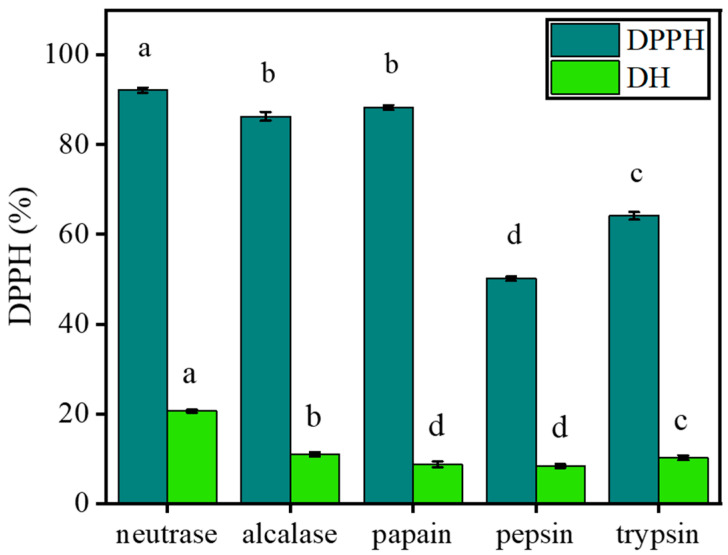
Degree of hydrolysis (DH) and 1,1-diphenyl-2-picrylhydrazyl (DPPH) scavenging ability of five proteases after hydrolysis. Data are represented as mean ± SD (*n* = 3). Letters a–d denote statistically significant differences among treatments (*p* < 0.05).

To obtain hydrolysates with high efficiency, we tried to screen the best protease for hydrolysis. For each reaction, the same solid/liquid ratio, enzyme volumes, and enzymolysis time were used with the optimal pH and temperature of each enzyme. DH and DPPH scavenging ability for the five hydrolysates were as follows: neutrase > alcalase > trypsin > papain > pepsin and neutrase > alcalase > papain > trypsin > pepsin (Figure 1). Therefore, neutrase was selected for subsequent modifications.

### 2.2. Single-Factor Experiments

**Figure 2:**
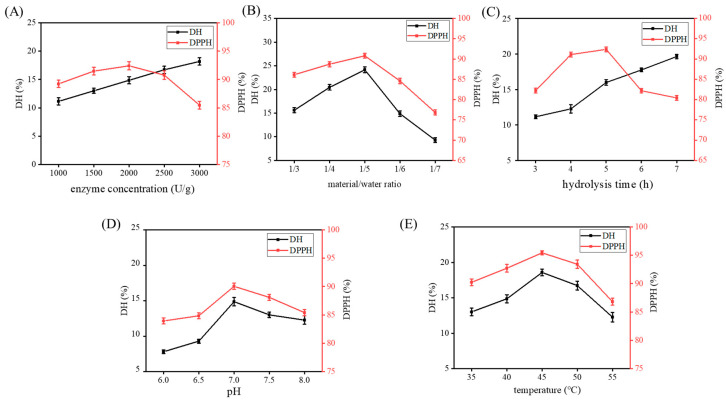
DPPH clearance rate and degree of hydrolysis (DH) vary with enzyme concentration (**A**), material/water ratio (**B**), hydrolysis time (**C**), pH (**D**), and temperature (**E**). Data are represented as mean ± SD (*n* = 3).

Figure 2 illustrates the influence of five single factors on DH and DPPH clearance rates. The DPPH clearance rate initially reached a maximum and then decreased. DH increased with an increase in enzyme dosage and time. In the other three single factor ranges, DH first increased and then decreased. The following conditions were determined to be ideal for extraction: enzyme concentration of 2000 U/g, material/water ratio of 1:5 (*w/v*), hydrolysis time of 5 h, pH of 7.0, and temperature of 45 °C.

### 2.3. Determination of Optimal Conditions Using RSM to Produce LPs by the Action of Neutrase

Three independent parameters at three levels were used in a Box–Behnken and Design (BBD) based on single-factor experiments. The optimal bromelain treatment conditions required to produce monkfish muscle protein hydrolysate was determined using RSM. Equations were used to evaluate the effects of different enzyme conditions on DH and DPPH. Material/water ratio (A), extraction time (B), pH (C), and a statistically significant interaction (*p* < 0.05) on the DH and DPPH value were explained as follows:

DH = 20.590 + 0.600A − 0.460B + 0.046C + 0.280AB + 0.370AC − 0.093BC − 3.790A^2^ − 1.840B^2^ − 3.42C^2^

DPPH = 92.700 + 0.890A + 0.820B − 0.250C + 0.460AB + 0.550AC + 1.200BC − 1.420A^2^ − 2.990B^2^ − 3.56C^2^

ANOVA results for the model are listed in Table 1 and Table 2. The corresponding parameters were more significant as the *F*-value increased and the *p*-value decreased [20]. Based on the analysis as seen in Table 1 and Table 2 *p* < 0.0001 and *p* = 0.0006 of the two models show that the two models are extremely significant; *p* = 0.1663 > 0.05 and *p* = 0.0571 > 0.05 of the disharmony term are not significant; the regression equations of the DH and DPPH free radical scavenging rate are significantly tested, and the correlation coefficients of the two models are *R*^2^ = 0.984 3 > 0.95 and *R*^2^ = 0.956 > 0.95, respectively. The coefficients of variation for DH and DPPH activities are 3.49% and 1.04%, respectively. It shows that the fitting degree of the model is good. It can be seen that the two models can better reflect real test values. It was found that factors A, A^2^, B^2^, and C^2^ had significant effects on DH (*p* < 0.05). Factors A, B, BC, A^2^, B^2^, and C^2^ had significant effects on the DPPH free radical scavenging rate (*p* < 0.05).

**Figure 3:**
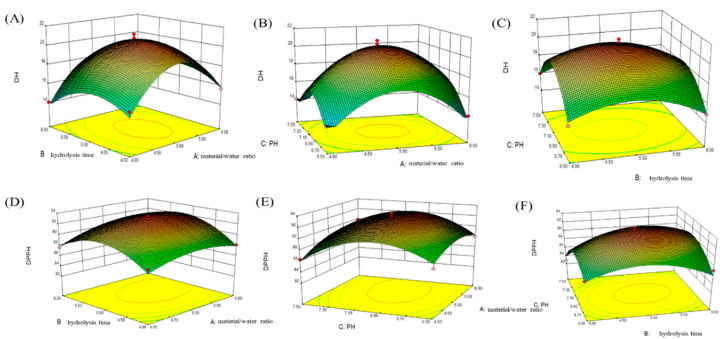
Response surface of hydrolysis rate (DH) and DPPH free radical scavenging rate of enzymatic hydrolysate, (**A**) the degree of hydrolysis (DH) is the response value of the interaction between hydrolysis time and material/water ratio, (**B**) degree of hydrolysis (DH) is the interaction of response value pH and material/water ratio, (**C**) degree of hydrolysis (DH) is the interaction of response value, hydrolysis time, and pH, (**D**) DPPH free radical scavenging rate is the interaction of response value, hydrolysis time, and material/water ratio, (**E**) DPPH radical scavenging rate is the interaction of response value, pH, and material/water ratio, (**F**) The scavenging rate of DPPH free radical is dependent on response value, and the interaction of hydrolysis time and pH value.

Each response surface was obtained using the Box–Behnken test regression model. According to the bending degree of the response surface, the experimental points in Figure 3 fall within the experimental conditions, indicating that the optimal conditions are in this range. Figure 3A–C shows the effects of the interaction of hydrolysis time and material/water ratio, pH and material/water ratio, and pH and hydrolysis time on DH. On the whole, the *p*-value of each interaction was greater than 0.05 (*p* = 0.3604, *p* = 0.2333, *p* = 0.7538), and the interaction was not significant. The dimension reduction analysis of each factor is the trend of increasing first and then decreasing. The response value is the highest in the middle region, and the DH is the highest. Figure 3D–F shows the effects of hydrolysis time and material/water ratio, pH and material/water ratio, and pH and hydrolysis time on DPPH. The results are similar to DH. The interaction between enzymatic hydrolysis time and material/water ratio, pH and material/water ratio is not significant, and the response value reaches the maximum in the middle region, and DPPH is the highest. However, the interaction of pH and hydrolysis time had a significant effect on the proportion of oligopeptides in the hydrolysate (*p* < 0.05).

According to the analysis of design expert v8.0.6 software with degree of hydrolysis (DH) as the response index, the optimal experimental conditions were determined as follows: water/material ratio of 5.67:1, a time span of 4.92 h, pH of 7.02; the predicted degree of hydrolysis (DH) was 19.322%. Taking the DPPH free radical scavenging rate as the response index, the optimal experimental conditions were as follows: water/material ratio of 5.35:1, a time span of 5.17 h, pH of 7.01; the predicted DPPH scavenging rate was 92.921%. Considering the maneuverability of the experiment, the optimal conditions were adjusted as follows: water/material ratio of 5.40:1, a time span of 5 h, and pH of 7.0. The results of three experiments under this condition showed that the degrees of hydrolysis (DH) were 19.293%, 19.302%, and 19.312%, respectively. The calculated average value was 19.302%, which was close to the predicted value; the DPPH radical scavenging rates were 92.893%, 92.731%, and 92.959%, respectively. The calculated average value was 92.861%, which was close to the predicted value. Taken together, the established model is superior.

### 2.4. MW Distribution of Monkfish Muscle Protein Hydrolysate

**Figure 4:**
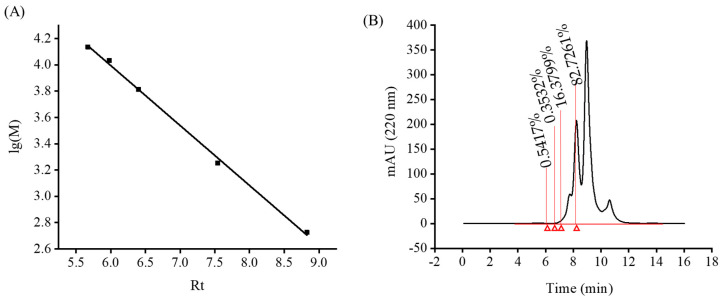
Relative molecular mass standard curve (**A**) and chromatogram of relative molecular mass distribution of monkfish muscle peptides (LPs) (**B**). Rt: retention time; lg(M): logarithm of MW.

In most cases, functional antioxidative peptides can only be activated by the process of hydrolysis, and most of them have small sizes that are less than 1 kDa [21,22]. The MW distribution of monkfish muscle peptides (LPs) was determined by a high-performance liquid chromatography (Agilent 1260, Palo Alto, CA, USA). A linear regression equation was made for the standard curve (Figure 4A): lg (*M*) = −0.4564x + 6.7339, *R*^2^ = 0.998. The MW distribution of LPs was determined using the regression equation. The relative MW distribution of monkfish muscle hydrolysate under the optimal enzymolysis conditions was small and mainly concentrated below 3000 Da, of which 82.726% was less than 1000 Da (Figure 4B). Therefore, it is concluded that LPs may have better antioxidant activity.

### 2.5. Interception of LPs with Different MW Using Ultrafiltration

**Figure 5:**
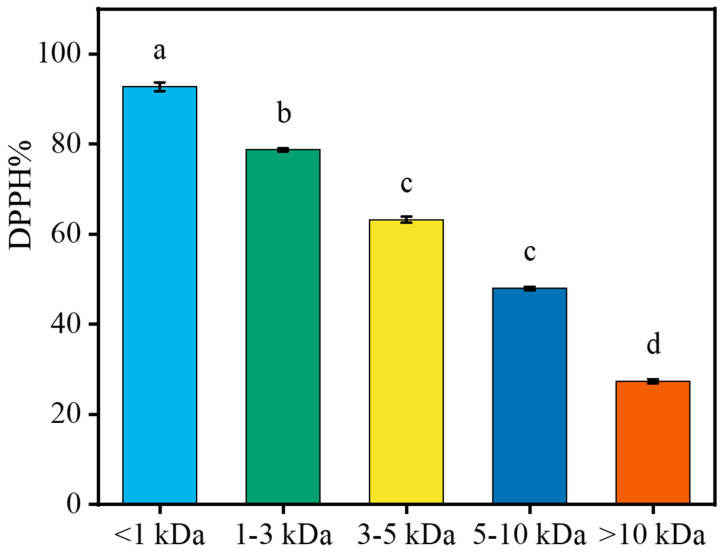
DPPH clearance of peptides with different molecular weights. Data are represented as mean ± SD (*n* = 3). Different superscript letters (a–d) denote statistically significant differences among treatments (*p* < 0.05).

DPPH is a type of stable free radical, and its response concentration is relatively low. It is usually used as a substrate to measure antioxidant activities [23]. Five different peptides were isolated from LPs using the membrane method. Figure 5 shows the DPPH radical scavenging activities of different LPs at 5 mg/mL. Low MW peptides were more effective than the high MW peptides in scavenging DPPH free radicals, especially the fraction <1 kDa that may have contained peptides/amino acids. As hydrogen donors, low MW peptides transform free radicals into more stable products, which show a higher DPPH-scavenging effect. This is consistent with the results of high DPPH scavenging activity of low MW peptides isolated from the protein hydrolysate of tuna by Hsu [24] and that from salmon hydrolysate by Ahn et al. [25].

### 2.6. Amino Acid Content of LPs <1 kDa

The hydrophobic nature of peptides helps elevate their antioxidant properties. They increase the interaction with lipid targets by virtue of hydrophobic associations or peptide entry into target organs, which are conducive to the realization of antioxidant effects [26,27]. Some studies have shown that negatively charged amino acids (NCAAS), such as glutamic and aspartic acids, exhibit strong antioxidant capacity due to a surplus of electrons that can be used during a free radical reaction [26,28,29]. As shown in Table 3, the percentage of hydrophobic amino acids (HAA) and NCAAS of <1 kDa LPs were relatively high, which were 25.74 (g/100 g) and 15.59 (g/100 g), respectively. The content of essential amino acids in <1 kDa LPs was 28.43 (g/100 g). We found that tryptophan, asparagine, and glutamine were not detectable due to the acidic conditions of analysis. These results indicated that <1 kDa LPs had appreciable antioxidant capacity and nutritional value.

### 2.7. Antioxidant Activity of <1 kDa LPs

**Figure 6:**
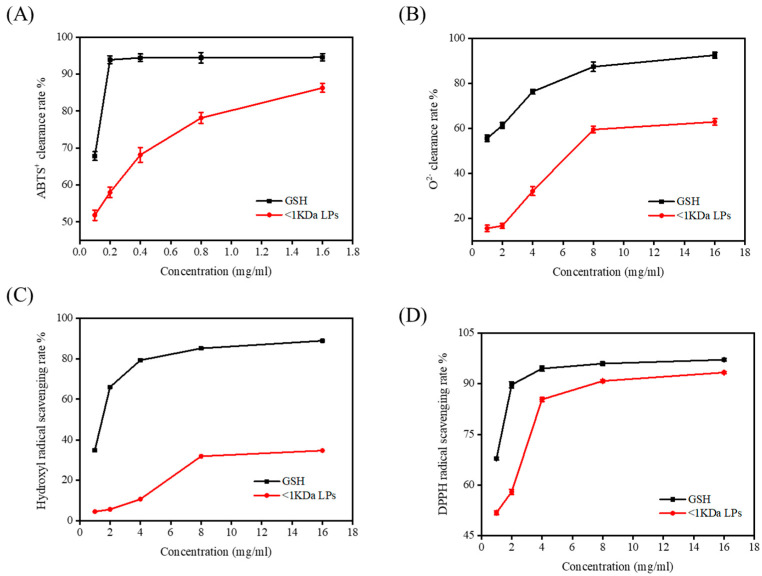
Scavenging effects of the <1 kDa LPs on 2,2′-azinobis-3-ethylbenzthiazoline-6-sulphonate (ABTS) radical (**A**), superoxide anion radical (**B**), hydroxyl radical (**C**), and DPPH radical (**D**). Data are represented as mean ± SD (*n* = 3).

LPs were purified using an ultrafiltration membrane with MW of 1000 kD, and <1 kDa LPs were obtained and freeze-dried. The clearance rates of 2,2′-azinobis-3-ethylbenzthiazoline-6-sulphonate (ABTS), superoxide anion, DPPH radical, and hydroxyl radical were determined (Figure 6). The antioxidant capacity of <1 kDa LPs increased with increasing concentrations and was therefore dose-dependent. Among them, the ABTS scavenging effect was strong and provided a theoretical basis for the development of functional foods with antioxidant effects by using monkfish resources.

### 2.8. Effect of <1 kDa LPs on the Viability of H_2_O_2_-Stimulated Cells

**Figure 7:**
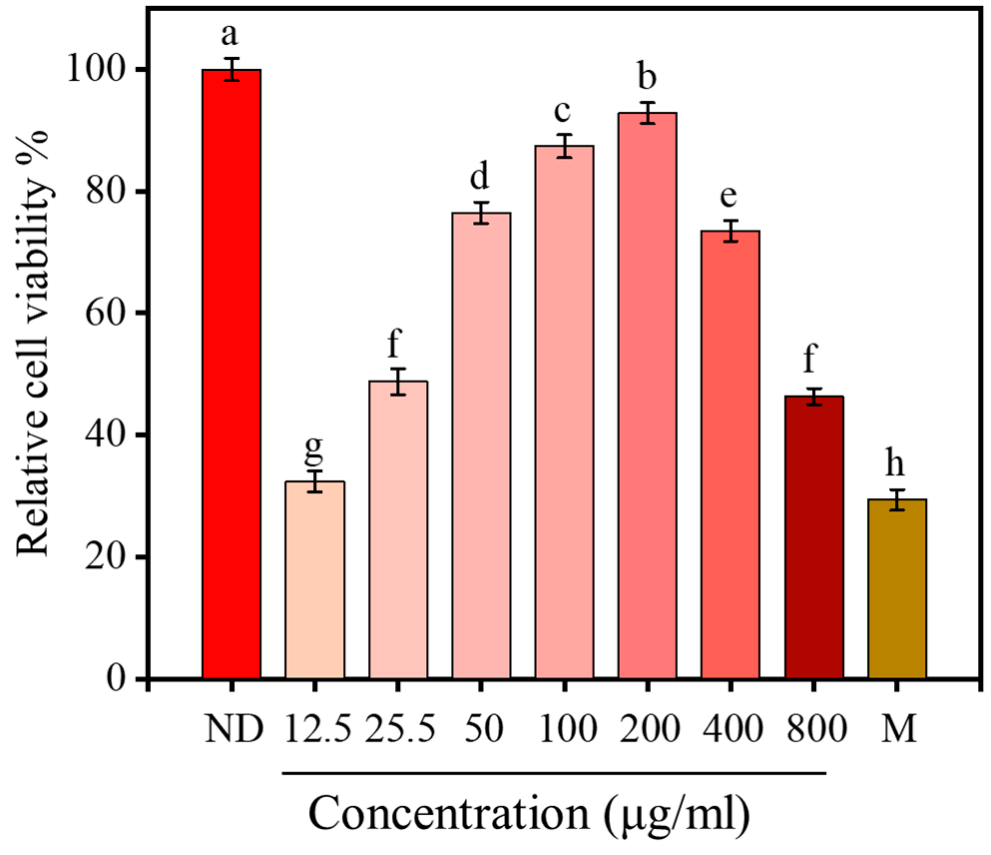
Protective effects of <1 kDa LPs on the viability of H_2_O_2_-stimulated cells. Results are expressed as means ± SD (*n* = 8). Letters a–h denote statistically significant differences among treatments (*p* < 0.05).

H_2_O_2_ treatment significantly reduced the viability of RAW264.7 cells (*p* < 0.05), which decreased to 29.20% after 12 h of H_2_O_2_ treatment, indicating that H_2_O_2_ stimulation could cause oxidative damage. After 12 h of pretreatment with <1 kDa LPs, the activity of macrophages significantly increased (*p* < 0.05) and showed a concentration-dependent effect (Figure 7). When the concentration of <1 kDa LPs reached 200 μg/mL, the activity of macrophages reached 92.29%. Therefore, the concentration of 50, 100, and 200 μg/mL were selected for further studies.

### 2.9. Effects of <1 kDa LPs on the ROS Levels

**Figure 8:**
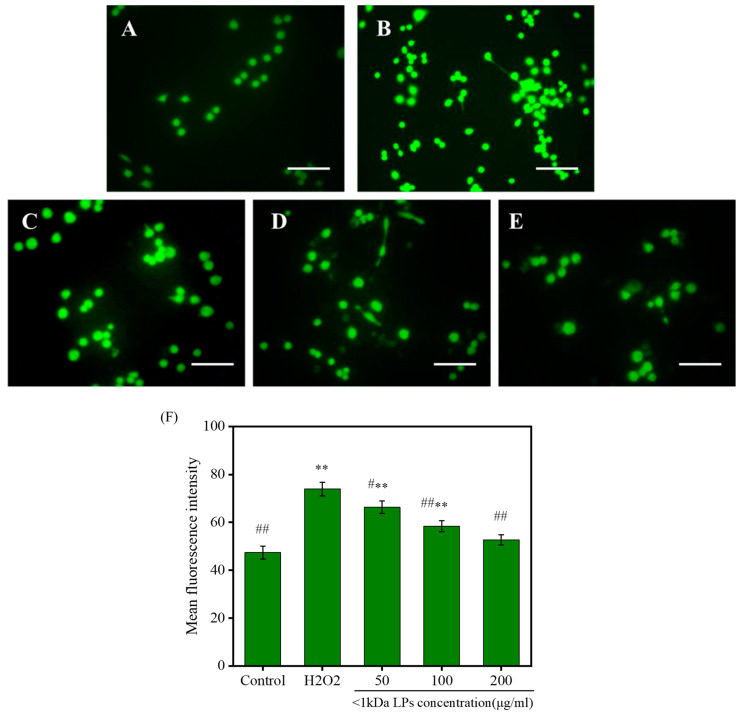
Inhibitory effects of <1 kDa LPs on intracellular reactive oxygen species (ROS) levels in H_2_O_2_-stimulated cells. (**A**) Control group; (**B**) model group (500 μM H_2_O_2_); (**C**) 50 µg/mL <1 kDa LPs+ 500 µM H_2_O_2_; (**D**) 100 µg/mL <1 kDa LPs+ 500 µM H_2_O_2_; (**E**) 200 µg/mL <1 kDa LPs+ 500 µM H_2_O_2_. (**F**) Mean fluorescence intensity of cells in different groups. Data are presented as the mean ± SD (*n* = 3). ** *p* < 0.01 vs. the control group, ^#^ *p* < 0.05, ^##^ *p* < 0.01 vs. the H_2_O_2_ group. Scale bars = 50 μm.

Reactive oxygen species (ROS) levels in the cells that were treated with <1 kDa LPs are shown in Figure 8. H_2_O_2_-treated cells showed higher fluorescence intensity, indicating that H_2_O_2_ treatment could significantly increase intracellular ROS levels. Compared to the cells treated with H_2_O_2_, the fluorescence intensity of the cells treated with <1 kDa LPs was lower than that of the cells treated with H_2_O_2_, which indicated that pretreatment with <1 kDa LPs could reduce the level of ROS in the cells stimulated by H_2_O_2_, and this effect showed a concentration-dependent effect. These results showed that <1 kDa LPs could act as a potential ROS scavenger.

### 2.10. Effect of <1 kDa LPs on Antioxidant Activities

**Figure 9:**
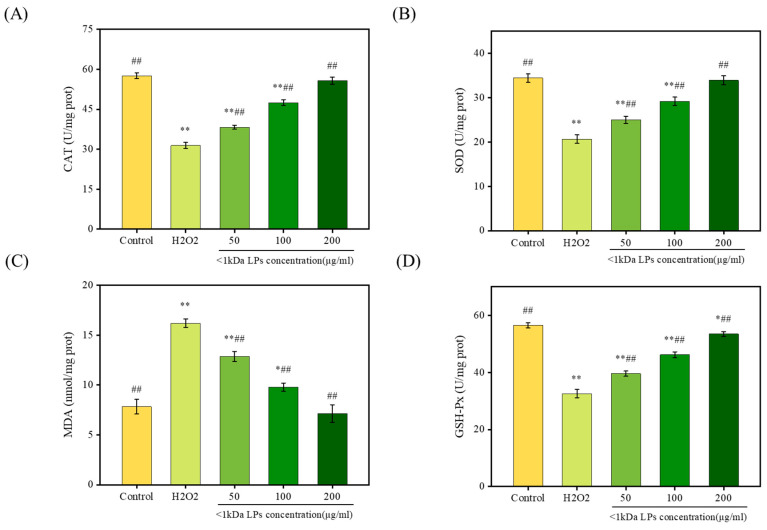
Effect of <1 kDa LPs on levels of catalase (CAT) (**A**), superoxide dismutase (SOD) (**B**), malondialdehyde (MDA) (**C**), and glutathione peroxidase (GSH-Px) (**D**) in H_2_O_2_-induced RAW264.7 injury model. Data are represented as mean ± SD (*n* = 3). * *p* < 0.05, ** *p* < 0.01 vs. control group, ^#^ *p* < 0.05, ^##^ *p* < 0.01 vs. H_2_O_2_ group.

Superoxide dismutase (SOD), catalase (CAT), and glutathione peroxidase (GSH-Px), as intracellular antioxidant enzymes, can inhibit free-radical attack on cell membranes and maintain cell health. Malondialdehyde (MDA) is an oxidative metabolite of lipid oxidation that can be used as a marker for lipid peroxidation and cell lesions [30]. To measure the antioxidant activity of <1 kDa LPs, we studied the effects of pretreatment with <1 kDa LPs on GSH-Px, SOD, CAT, and MDA levels after H_2_O_2_ injury. H_2_O_2_ significantly reduced the levels of SOD, CAT, and GSH-Px, indicating that oxidative stress induced a serious damage to the antioxidant enzymes (Figure 9). The levels of SOD, CAT, and GSH-Px in cells that were pretreated with <1 kDa LPs increased dose-dependently, which were significantly higher than those in the H_2_O_2_-injured cells (*p* < 0.01). Compared to the control group, the MDA level in cells treated with H_2_O_2_ was markedly increased (*p* < 0.01), indicating that H_2_O_2_ was responsible for damage to these cells. Therefore, <1 kDa LPs could reduce oxidative stress injury and abate lipid peroxidation. The exact mechanism of how LPs work inside the cell to reduce oxidative stress needs to be thoroughly studied by analyzing the expression of antioxidant enzymes in the follow-up studies.

## 3. Materials and Methods

### 3.1. Pretreatment of Monkfish (Lophius litulon) Muscle

Monkfish was purchased from the Pearl fish market in Zhoushan, Zhejiang. Muscles were separated from the bones and then ground. Degreasing was carried out with 95% ethanol (1:5, *w/v*) in a water bath (50 °C) for 2 h [31]. The supernatant was discarded, and the precipitate was washed with distilled water. The defatted muscle was centrifuged at 12,000 r/min for 15 min, and the precipitate was collected.

### 3.2. Optimization of Preparative Conditions of LPs

Trypsin (≥250 U/mg), neutrase (≥60 U/mg), alcalase (≥200 U/mg), pepsin (≥3000 U/mg), and papain (≥500 U/mg) were ordered from YTHX Biotech (Beijing, China). The defatted muscle was added with distilled water (1:5, *w/v*) and protease (2000 U/g). The pH and temperature were adjusted to the appropriate value of protease (Table 4) and then hydrolyzed for 5 h. Enzymes were inactivated at 100 °C for 10 min and centrifuged at 12,000 r/min for 10 min. A portion of the supernatant was used for determining the degree of hydrolysis (DH), while the rest was freeze-dried for determining free radical activities.

DPPH, ABST, and phenazine methosulfate (PMS) were purchased from Sigma-Aldrich (Shanghai, China). According to the results of DPPH and DH, neutrase was selected as the best protease. We selected five parameters (protease concentration, pH, temperature, hydrolysis time, and muscle/water ratio) to conduct single-factor experiments using DPPH radical scavenging ability and DH as indicators. Based on single-factor experiments and three other factors (the extraction time, muscle/water ratio, and pH), a three-level and three-factor response surface test was designed using the Box–Behnken and Design (BBD) Expert (Design-Expert^®^ 8.0). The test factors and levels are shown in Table 5. The antioxidant activities of the extracted peptides were evaluated using the degree of hydrolysis and DPPH clearance rate. Response surface test design and results are shown in Table 6. Responses were analyzed using multiple regressions to fit the following equation:

(1) $$\gamma =\beta_{0}+\sum_{i=1}^{k}\beta_{i}X_{i}+\sum_{i=1}^{k}\beta_{ii}X_{i}^{2}+\sum \sum_{i<j}\beta_{ij}X_{i}X_{j}$$

where *γ* is the predicted response, *β*_0_ is the intercept, *β_i_*, *β_ii_*, and *β_ij_* are the linear, quadratic, and interaction coefficients, respectively, and both *X_i_* and *X_j_* are independent factors.

Using the design expert 8.0, an analysis of variance table was compiled. The influence coefficient, the regression coefficient of the linear term, quadratic term, and interactive term were determined.

### 3.3. Determining the MW Distribution of LPs

The MW distribution of LPs was analyzed using HPLC (Agilent 1260, Palo Alto, CA, USA). To make the mobile phase, 55% (*v/v*) water, 15% (*v/v*) acetonitrile, and 0.1% trifluoroacetic acid were mixed [32]. The flow rate was 0.5 mL/min, and the column was calibrated using cytochrome C (12,400 Da), aprotinin (6511.44 Da), and bacitracin (1422.69 Da) (Songon Biotech, Shanghai, China), ribonuclease A (13,700 Da) (Fushen Biotech, Shanghai, China), and *Anthopleura anjunae* oligopeptide (Tyr-Val-Pro-Gly-Pro, 531.61 Da) [33]. According to the relationship between the logarithm molecular weight and elution time, the molecular weight distribution of LPs was calculated.

### 3.4. DH and Antioxidant Activity

DH was titrated using the method by Noman et al. with some modifications [34]. Five milliliter protein hydrolysate was mixed with 50 mL of distilled water and the pH was adjusted to 8.2 with 0.05 M NaOH. Formaldehyde solution (10 mL) was added, and the hydrolysate was subsequently placed at 25 °C for 5 min. Then, the mixture was titrated with 0.05 M NaOH at pH 9.2. The content of the free amino group was determined based on the volume of NaOH used. The total nitrogen content was calculated using the Kjeldahl method [35]. The free amino content and DH were analyzed using the following equations:

(2) $$\%\;\mathrm{free}\;\mathrm{amino}\;\mathrm{groups}=\frac{A\times B\times 0.014}{C/D}\times 100\%$$

(3) $$\mathrm{DH}=\frac{\%\;\mathrm{Free}\;\mathrm{amino}\;\mathrm{groups}}{\%\;\mathrm{total}\;\mathrm{nitrogen}}\times 100\%$$

where A = mL of NaOH used; B = the concentration of the solution used for titration (0.05 M NaOH); C = volume of sample diluent (mL); D = constant volume of sample (mL).

The DPPH, hydroxyl, ABTS, and superoxide anion radical scavenging activities of LPs <1 kDa were performed using the methods described by Chen et al. [36].

### 3.5. Preparation of LPs with Different Molecular Weight

The muscle hydrolysate was purified using a series of ultrafiltration membranes and a separation system (GM-18, Bona Biotech, Jinan, China) with a MW cut-off of 10, 5, 3, and 1 kDa. This process generated five parts as follows: >10 kDa, 5–10 kDa, 3–5 kDa, 1–3 kDa, and <1 kDa. The fractions were freeze-dried, and components with the best DPPH scavenging activity were selected. Amino acid content was determined with the method described by Tang et al. [37]. LPs were hydrolyzed with 6 mol/L HCl in an oven at 110 °C for 24 h. Approximately 1 mL of the sample was dried by blowing nitrogen, dissolved in 1 mL buffer solution (pH = 2.2), and measured by an amino acid analyzer (Hitachi L-8800, Tokyo, Japan).

### 3.6. Effects of <1 kDa LPs on theH_2_O_2_-Injured Cells

Mouse RAW264.7 cells were purchased from the Cell Bank of the Chinese Academy of Sciences (Shanghai, China). Cells were and seeded in 96-well plates at a density of 5 × 10^4^ cells per well. After culturing for 24 h, the medium was gently aspirated and replaced with LPs <1 kDa at different concentrations (12.5, 25, 50, 100, 200, 400, 600, 800, and 1000 μg/mL). Cells were cultured in the medium for 24 h and treated with H_2_O_2_ (final concentration of 500 μmol/L) for 8 h [38]. Then, 20 μL of 3-(4,5-dimethylthiazol-2-yl)-2,5-diphenyltetrazolium bromide (MTT) was added into the culture solution. The medium was gently aspirated from the plate, and 100 μL of dimethyl sulfoxide was added into each well. The absorbance was detected at 490 nm. The cell viability was determined as the absorbance of LPs group divided by the absorbance of control group. The cell viability was calculated using the following formula:

(4) $$\mathrm{Cell}\;\mathrm{viability}\;(\%)=\frac{A1}{A2}\times 100\%$$

where A1 is the absorbance of the LPs group and A2 is the absorbance of the control group.

### 3.7. Determination of ROS Levels after H_2_O_2_ Treatment

Cells were seeded in 6-well plates at a density of 2 × 10^5^ cells per well. After culturing for 24 h, cells were incubated with LPs for another 24 h. H_2_O_2_ (500 μmol/L) was added for 8 h. The medium was discarded and cells were rinsed with PBS three times. The fluorescent probe DCFH-DA solution (150 μL; 50 μmol/L) was added and maintained at 37 °C for 60 min. Subsequently, cells were rinsed with PBS three times [39]. Cells were visualized using fluorescence microscopy and photographed. The fluorescence intensity was analyzed using Image J software.

### 3.8. Antioxidant Enzyme Activity of <1 kDa LPs in H_2_O_2_-Induced Stress

First, 500 μL cell lysis buffer was added to each well, and the mixture was centrifuged at 12,000× *g* at 4 °C for 10 min. The supernatant was cooled to 4 °C. The levels of GSH-Px, SOD, CAT, and MDA were measured with commercial test kits (Nanjing Jiancheng Bioengineering Institute, Nanjing, China).

### 3.9. Statistical Analysis

The data were presented as the mean ± SD. Multiple-group comparisons were determined using ANOVA in SPSS 19.0. *p* = 0.05 was considered as a threshold with statistical significance.

## 4. Conclusions

In this study, the extraction procedure of antioxidative peptides from monkfish muscle were optimized. LPs less than 1 kDa had a high antioxidant activity and could effectively scavenge DPPH, hydroxyl, ABTS, and superoxide anion radicals. LPs possess good antioxidant capacity and protect RAW264.7 cells from H_2_O_2_-induced injury, which provides a theoretical basis for the use of <1 kDa LPs as natural antioxidants. Further studies are required to determine the peptide sequence of <1 kDa LPs and verify the protective functions in animals. Our optimized procedures for extracting LPs may also be useful for future scale-up production.

## Figures and Tables

**Table 1:** Analysis of variance of regression equation for degree of hydrolysis (DH).

Variables	Sum of Squares	*df*	Mean Square	*F*-Value	*p*-Value
A (material/water ratio)	2.92	1	2.92	8.99	0.0200
B (extraction time)	1.73	1	1.73	5.32	0.0545
C (PH)	0.017	1	0.017	0.053	0.8242
AB	0.31	1	0.31	0.96	0.3604
AC	0.55	1	0.55	1.70	0.2333
BC	0.035	1	0.035	0.11	0.7538
A^2^	60.52	1	60.52	186.41	<0.0001
B^2^	14.25	1	14.25	43.90	0.0003
C^2^	49.23	1	49.23	151.65	<0.0001
Model	142.64	9	15.85	48.82	<0.0001
Lack of fit	4.02	7	0.57		
Residual	1.55	3	0.52	2.88	0.1663
Pure error	0.72	4	0.18		
Cor. total	144.91	16			
*R*^2^ = 0.9843		*R_Adj_*^2^ = 0.9642		CV = 3.49%	

**Table 2:** Analysis of variance of regression equation for degree of hydrolysis (DPPH).

Variables	Sum of Squares	*df*	Mean Square	*F*-Value	*p*-Value
A (material/water ratio)	5.43	1	5.43	6.40	0.0291
B (extraction time)	6.35	1	6.35	7.48	0.0393
C (PH)	0.48	1	0.48	0.57	0.4757
AB	0.84	1	0.84	0.99	0.3539
AC	5.78	1	5.78	6.80	0.2701
BC	1.22	1	1.22	1.43	0.0350
A^^2^	37.56	1	37.56	44.24	0.0161
B^^2^	8.43	1	8.43	9.93	0.0003
C^^2^	53.45	1	53.45	62.95	<0.0001
Model	129.48	9	14.39	16.95	0.0006
Lack of fit	5.94	7	0.85		
Residual	4.87	3	1.62	6.06	0.0571
Pure error	1.07	4	0.27		
Cor. total	135.42	16			
*R* ^2^ * = 0.9561*		*R_Adj_* ^2^ * = 0.8997*		CV = 1.04%	

**Table 3:** Amino acid composition (g/100 g) of <1 kDa LPs.

Amino Acids	LPs
Asp	5.82 ± 0.190
Thr	2.83 ± 0.083
Ser	2.89 ± 0.080
Glu	9.75 ± 0.297
Gly	2.53 ± 0.080
Ala	3.45 ± 0.107
Cys	0.00
Val	3.51 ± 0.096
Met	2.42 ± 0.051
Lle	2.79 ± 0.079
Leu	5.57 ± 0.152
Tyr	1.81 ± 0.014
Phe	6.19 ± 0.079
Lys	5.48 ± 0.167
His	1.55 ± 0.102
Arg	3.70 ± 0.105
Pro	0.00
HAA	25.74
PCAA	10.73
NCAA	15.59
EAA	28.43

**Table 4:** Optimal reaction conditions of proteases.

Types of Protease	Optimum Temperature (°C)	Optimum pH
neutrase	45 °C	7.0
alcalase	45 °C	10.0
papain	60 °C	6.0
pepsin	37 °C	2.0
trypsin	37 °C	8.0

**Table 5:** Box–Behnken test factors and levels.

Variables	Levels and Range		
	−1	0	1
A muscle/water ratio (g·mL^−1^)	1:4	1:5	1:6
B extraction time (h)	4	5	6
C pH	6.5	7	7.5

**Table 6:** Response surface test design and results.

Run Numbers	A Extraction Time (h)	B Muscle/Water Ratio (g/mL)	C PH	DH%	DPPH/%
1	−1	0	−1	13.0088	86.7955
2	0	0	0	20.4425	93.4659
3	0	−1	−1	15.2389	86.9091
4	−1	0	1	11.8939	86.375
5	0	0	0	20.0708	92.1818
6	1	−1	0	15.6106	88.1136
7	0	0	0	20.8142	92.4659
8	0	1	−1	14.8673	87.0568
9	0	0	0	21.1858	92.4091
10	−1	−1	0	15.6106	87.7386
11	1	0	1	14.4956	89.752
12	1	1	0	14.8673	89.7727
13	−1	1	0	13.7522	87.5682
14	1	0	−1	14.1239	87.9659
15	0	1	1	15.2389	87.7955
16	0	−1	1	15.9823	82.841
17	0	0	0	20.4425	92.9773
